# Emerging Roles of Osteoclasts in the Modulation of Bone Microenvironment and Immune Suppression in Multiple Myeloma

**DOI:** 10.3389/fimmu.2017.00954

**Published:** 2017-08-11

**Authors:** Anna Mansour, Abdelilah Wakkach, Claudine Blin-Wakkach

**Affiliations:** ^1^CNRS, UMR7370, LP2M, Faculté de Médecine, Nice, France; ^2^Université Nice Sophia Antipolis, Nice, France; ^3^Faculté de Médecine, Université Aix-Marseille, Marseille, France

**Keywords:** osteoimmunology, osteoclast, multiple myeloma, myeloma bone disease, immunomodulation, hematopoietic niche

## Abstract

Multiple myeloma (MM) is one of the most common forms of hematologic malignancy resulting from cancerous proliferation of mature malignant plasma cells (MPCs). But despite the real improvement in therapeutics in the past years, it remains largely incurable. MM is the most frequent cancer to involve bone due to the stimulation of osteoclast (OCL) differentiation and activity. OCLs have a unique capacity to resorb bone. However, recent studies reveal that they are not restrained to this sole function. They participate in the control of angiogenesis, medullary niches, and immune responses, including in MM. Therefore, therapeutic approaches targeting OCLs probably affect not only bone resorption but also many other functions, and OCLs should not be considered anymore only as targets to improve the bone phenotype but also to modulate bone microenvironment. In this review, we explore these novel contributions of OCLs to MM which reveal their strong implication in the MM physiopathology. We also underline the therapeutic interest of targeting OCLs not only to overcome bone lesions, but also to improve bone microenvironment and anti-tumoral immune responses.

## Introduction

Representing 13% of hematologic and 1% of all the cancers (1), multiple myeloma (MM) is the most common form of hematologic malignancy after non-Hodgkin lymphoma and the most frequent cancer to involve bone (2). MM is a plasma cell neoplasm of complex etiology, resulting from a cancerous proliferation of mature malignant plasma cells (MPCs) and characterized by the production of monoclonal intact immunoglobulins or immunoglobulin free light or free heavy chains. MM arises from the interaction between adverse environmental and inherited genetic risk factors (3). While the therapy of MM has greatly improved in the past 15 years, its prognosis remains poor and a better understanding of the mechanisms involved in the disease is still needed.

Genetic analysis has demonstrated that the pre-MPCs share some of the mutations associated with MM and that the disease progresses through multiple genetic waves of MM cell clones (3, 4). Indeed, during the course of the disease, the clonal composition of MPCs changes while they acquire further genetic abnormalities. These changes result in patients in the presence of multiple subclones genetically distinct (5–7) that further expand or compete for stromal niches within the bone marrow (BM) in a landscape that is continually changed by therapies (4).

Malignant plasma cells originate from the lymph nodes and migrate to the BM where they localize in contact with BM stromal cells (8, 9). The interaction of MPCs with BM stromal cells is crucial for their homing, survival, and proliferation and results in secondary pathologic conditions, such as bone destruction by osteoclasts (OCLs) recruited around MPCs (10). This bone destruction stimulates tumor growth through the release of growth factors from the bone matrix and results in severe bone pain and pathological fractures in the vast majority of patients presenting with MM. It also causes hypercalcemia, immunosuppression, and increased susceptibility to infections (11).

Osteoclasts are multinucleated giant cells of hematopoietic origin having a unique capacity to resorb the bone matrix. Recent studies, including from our group, have provided new finding demonstrating that OCLs are not only bone-resorbing cells but they are also involved in broader functions. OCLs regulate BM niches for hematopoietic stem cells (12, 13), B cell progenitors (14), and MPCs (15). They are capable of driving immune T cell response toward immunosuppression or inflammation according to their origin and to their environment (16–19), and participate in maintaining an immune suppressive environment in MM conditions (20).

Hence, because of these emerging pieces of evidence that OCLs may participate in the physiopathology of MM by modulating the immune response and the preservation of MPCs in the BM, their full clinical impact has yet to be more deeply evaluated. This review addresses this newly identified contribution of OCLs to MM, in particular their interactions with MPCs and their immunomodulatory function. It brings a more precise knowledge of the implication of OCLs in the physiopathology of MM that leads to consider targeting of OCLs as a complementary approach to improve MM therapy (21).

## Myeloma Bone Disease

Multiple myeloma evolves through various distinct clinical phases, including asymptomatic monoclonal gammopathy of undetermined significance (MGUS) and smoldering multiple myeloma (SMM; also known as asymptomatic myeloma). Presence of MPCs cells in the BM is a hallmark of MM. These cells are arising from the B cell lineage. They are complex cells with a morphological, cytogenetical, and phenotypic diversity, characterized by the expression of different markers. As normal plasma cells, MPCs express CD38 and CD138. CD138 (syndecan-1) is a proteoglycan that binds growth factors, chemokines, cytokines, and extracellular matrix components and regulates the interactions between MPCs and their environment (22, 23). CD38 is a transmembrane glycoprotein having the ability to interact with CD31 and having ecto-enzyme activities. It is involved in the regulation migration, adhesion, activation, and survival of leukocytes, including normal and MPCs (22, 24), as well as in bone remodeling (25) as described below, making it a very good therapeutic target (24). Myeloma cells are also characterized by the expression of SLAMF7, a glycoprotein expressed in hematopoietic cells, such as NK or CD8^+^ T cells and overexpressed by MPCs (26, 27). SLAMF7 plays an important role in the interaction between myeloma cells and BM stromal cells.

Multiple myeloma is characterized by the development of bone lytic lesions in up to 90% of the patients resulting in myeloma bone disease (2). In the BM, interaction between MPCs and BM stromal cells induces an alteration of the dynamic balance between bone formation and bone resorption. It results in an increased OCL differentiation and activity and a decreased osteoblast (OBL) number leading to the development of bone lesions that could extend from discrete lytic lesions to osteopenia affecting any part of skeleton, but preferably the spine, skull, and long bones (28). By the end of the nineteenth century, the first description of MM was a disease making bones deficient and fragile and “multiple” referred to several bones affected at a time. These osteolytic lesions were identified by X-rays in 1903 by Weber and whole body skeletal radiography remained the traditional gold standard for identification of lytic bone lesion. However, it does not detect early bone lesions and underestimates the extent of bone involvement (29) and low-dose computed tomography, magnetic resonance imaging, and positron emission tomography with computed tomography are now recommended for a more sensitive evaluation of myeloma patients (30, 31).

## OCL Activation in Myeloma

Multiple myeloma is the most frequent cancer to involve skeleton with up to 90% of patients developing bone lesions affecting the axial and appendicular skeleton during the disease course (32). MM symptoms are dominated by bone marks, such as bone pain (70–80% of the patients), fractures (50–60%), hypercalcemia (15%), and spinal cord compression (2–3%). Osteolytic lesions are a major cause of morbidity, decreased quality of life, poor mobility, and possible mortality (33).

**Figure 1:**
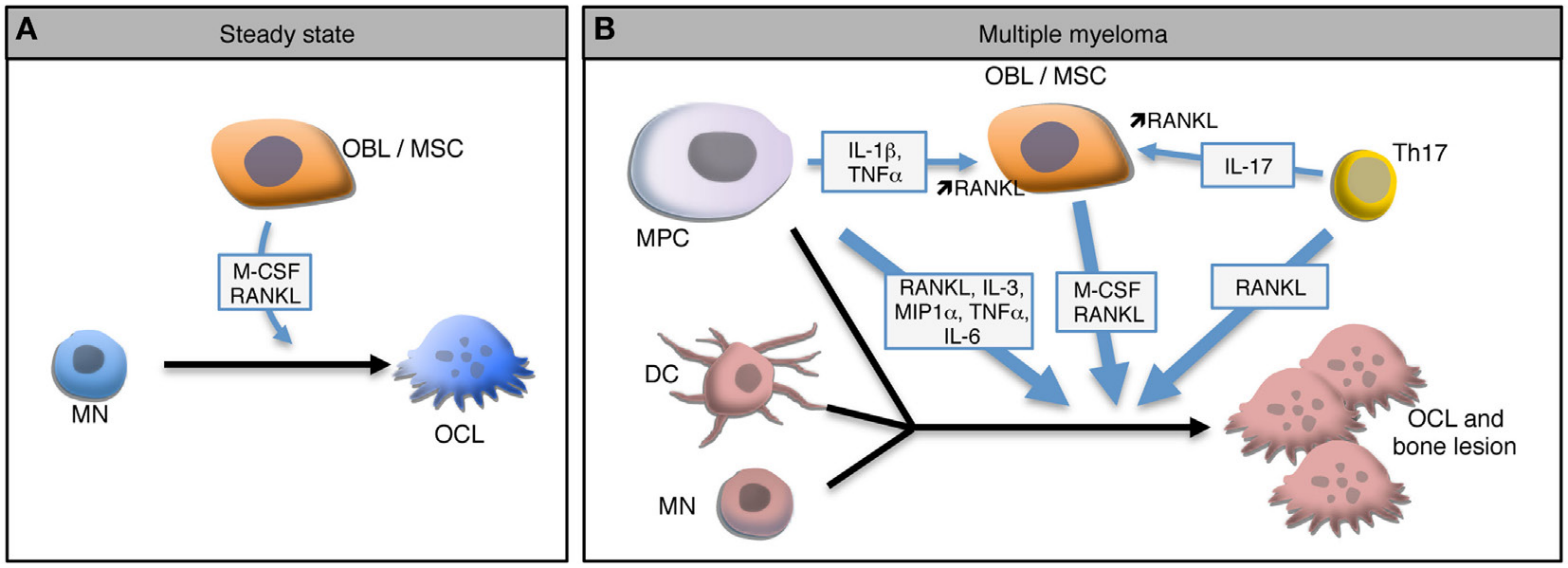
Osteoclast (OCL) development in steady state and multiple myeloma (MM). **(A)** In steady state, osteoclasts (OCLs) derive from monocytes (MN) under the influence of M-CSF and RANKL produce by osteoblasts (OBLs) and mesenchymal stromal cells (MSCs). **(B)** In MM environment, malignant plasma cells (MPC) produce and stimulate the production of RANKL by MSCs leading to a higher OCL differentiation. In the presence of such high levels of RANKL and IL-17 produced by Th17 cells, OCLs arise not only from MNs but also from dendritic cells (DCs). Moreover, MPCs form OCL-like polycaryons in the presence of high RANKL levels. Combination of these mechanisms lead to a dramatic increase of OCLs and to bone lesions.

This bone destruction is due to the over-activation of OCLs, the main if not the exclusive cells specialized in bone resorption. OCLs are multinucleated giant cells derived from monocytic precursor cells, mainly monocytes (MNs). In steady state, OCL differentiation requires close interactions with BM mesenchymal stromal cells (MSCs) and bone-forming OBLs that produce the two main factors required for OCL differentiation: macrophage-colony stimulating factor (M-CSF) and receptor activator of NFκB (RANK) ligand (RANKL) (Figure 1A) (34, 35). M-CSF is involved at early stages of OCL differentiation by playing a role in the survival and proliferation of OCL precursors. Interaction between RANK expressed on pre-OCLs and RANKL expressed on MSCs, OBLs, and osteocytes plays a key role in the differentiation, fusion, survival, and activity of OCLs, whereas osteoprotegerin (OPG), a decoy receptor for RANK ligand secreted mainly by stromal cells, inhibits RANK-RANKL signaling (35–38). Active OCLs are characterized by a very important extra- and intra-cellular proteolytic activity and a secretion of protons necessary for the degradation of the bone matrix (39). Bone resorption releases from the bone matrix growth factors that activate OBL differentiation, such as TGFβ, BMP2, IGF, or PDGF (40). Hence, in steady state, OCL and OBL differentiation and activity are tightly coupled to maintain bone homeostasis and integrity (40). However, under pathological conditions related to bone destruction, this coupling is altered and the expression of RANKL and M-CSF is dramatically increased mainly due to the production of inflammatory cytokines by immune cells, in particular CD4^+^ T cells. Pro-inflammatory cytokines, such as IL-1β, IL-6, TNF-α, and IL-17, can positively influence this osteoclastic differentiation directly (41, 42) or through OBLs (43) or Th17 lymphocytes to increase RANKL production (Figure 1B) (44–46).

As in inflammatory conditions, the coupling between OCLs and OBLs is altered in MM. Myeloma cells interact with other cells of the bone microenvironment to increase their survival and proliferation which also results in other pathologic conditions such as bone destruction by the OCLs recruited around myeloma cells (10). But contrasting with metastatic tumors such as breast and prostate cancers in which both the osteoclastic and osteoblastic activities are increased, no bone formation is observed in MM (47). Indeed, myeloma bone disease is related not only to OCL hyperactivity but also to an inhibition of OBL function (48). Comparative transcriptomic analysis revealed that the Wnt inhibitor Dickkopf 1 known to block OBL differentiation is highly expressed in MPCs from MM patients with bone lesion compared to normal plasma cells or plasma cells from MM patients without bone lesions, suggesting that inhibition of OBL differentiation in MM is also mediated by MPCs (10).

Regarding OCLs, myeloma cells trigger a coordinated upregulation in RANKL and decrease in OPG expression by BM MSCs and OBLs leading to an increased OCL differentiation and activity in patients (49–52). MPCs express RANKL (53) and interact with MSCs through the VLA-4d integrin to stimulate their production of RANKL (Figure 1B) (50). Several other factors that are upregulated during MM have been shown to contribute to OCL formation independently from RANKL. Macrophage inflammatory protein-1 alpha (MIP-1 alpha) and IL-3 are overexpressed in the BM of MM patients in correlation with the severity of the disease and induce OCL differentiation *in vitro* (54–56). Interestingly, the effect of IL-3 has been shown to be mediated by the production of Activin A by CD14^+^ MNs (57). In addition to increase osteoclastogenesis, this mechanism participates in the decrease of OBL formation (57). Blocking of Activin A in a humanized murine model of MM ameliorates the bone phenotype and inhibits tumor growth (58).

The MM BM environment not only provides a dramatic increase in osteoclastogenic factors but also favors the recruitment of various OCL progenitors. In conditions of very high RANKL production, the differentiation of OCLs differs from steady state since OCLs not only differentiate from MNs but also from dendritic cells (DCs) (Figure 1B). In 2004, Rivollier et al. reported for the first time the *in vitro* differentiation of human DCs generated from circulating blood MNs toward mature OCLs under M-CSF and RANKL stimulation and in the presence of synovial fluid from arthritic patients (59). This differentiation pathway has also been reported *in vivo* where it requires the presence of CD4^+^ T cells producing IL-17 and responsible for a high RANKL expression (60). This differentiation pathway arises from different DCs subsets: immature DCs generated *in vitro* (19, 59), conventional splenic MHC-II^+^ CD11c^+^ DCs and even DCs matured in the presence of LPS or CpG (60). Nevertheless, not all DC subtypes share the same plasticity, since conventional DCs have a higher potential for generating mature OCs than plasmacytoid DCs (60). The DC-derived OCLs probably represent an important pool of OCLs in inflammatory conditions (19, 61). Interestingly, the differentiation of OCLs from DCs has also been reported in MM (Figure 1B). In myeloma, BM resident DCs recruit CD4^+^ T cells and prime Th17 differentiation (62). Presence of Th17 cells in the BM is associated with increased OCL differentiation (45) in particular from DCs (60). Moreover, in MM patients, the proportion of Th17 cells is correlated with the severity of bone lesions and *in vitro*, IL-17 stimulates the differentiation of bone-resorbing OCLs not only from BM cells (63) but also from DCs from MM patients (64).

Several studies have suggested that, in addition of providing osteoclastogenic factors, MCPs could participate more directly in bone resorption. Murine MM cells have been reported to have the capacity to resorb bone *in vitro* (65). After long-term culture, human myeloma cell lines generate adherent polycaryons that express OCL markers, such as tartrate-resistant acid phosphatase and calcitonin receptor, and are able to resorb mineralized matrix (66). These observations were further supported by a study showing that OCLs from MM patients contain nuclei baring translocated chromosome originating from MPC clones, suggesting that MCP can directly contribute to OCL formation in MM patients (67).

These data highly suggest that the combination of an overexpression of osteoclastogenic factors and the recruitment of various OCL precursors participate in the increased OCL formation and bone lesions in myeloma.

## OCLs and Myeloma Cell Niches

Myeloma cells have a tropism for the bone medullary compartment. The BM structure is complex and comprises multiple cell types, including MSCs and their derivatives, endothelial cells, neuronal cells, immune cells, and hematopoietic stem and progenitor cells (HSPCs) (68). The BM provides specialized environments known as niches that maintain HSPCs, control their fate, and the balance between their dormancy and proliferation thanks to the expression of growth factors, chemokines, adhesion molecules, and transmembrane ligands, as well as extracellular matrix components (68). Two main HSC niches have been defined for HSCs, the endosteal niche located close to the trabecular bone and involving osteoblastic cells, and the perivascular niche. However, the endosteal region is highly vascularized making difficult to clearly identified the exact contribution of each of these niches (69).

In addition, a number of cell types participate in the niches and their regulation, including OCLs (68, 70). In osteopetrotic mice lacking active OCLs, HSCs do not colonize the BM because of defective niches characterized by an impaired OBL differentiation and a decreased expression of the main niche factors (13). Restoration of OCL activity is sufficient for recovering OBL differentiation, functional niches, and HSC homing in the BM (13). Equivalent mechanisms were also involved in the niches for B cell progenitors (14). Blocking of OCL activity also modulates BM plasma cell niches (71). Moreover, bone-resorbing OCLs have been identified as regulators of HSPC mobilization under stress conditions (12). Stress-activated OCLs over produce proteolytic enzymes that inactivate some of the signals involved in stem cell anchorage and retention participating to HSPCs mobilization (12).

Bone marrow niches are not only involved in normal hematopoiesis but also in maintaining cancer cells, including malignant hematopoietic cells. Alsayed et al. have showed that, as for HSCs, homing of MM cells to the BM is dependent on the expression of SDF-1 by OBLs and MSCs and CXCR4 on MM cells (72). Intravital microscopy analysis in mice has demonstrated that, once in the BM, part of MCPs are maintained as dormant cells in close proximity to the endosteal niche region. These cells are more tumorigenic than other MCPs and are resistant to anti-cancer therapies (15, 73). Moreover, activation of OCLs by RANKL injection results in a release of MCPs from the endosteal region and a decreased number of dormant MCPs in the BM, indicating that OCL control reactivation of dormant myeloma cells by remodeling the endosteal niche (15) as described for HSCs (12).

The vascular niche is also important for MPCs. Angiogenesis is an essential step in tumor progression and malignant cells require vascularization to survive and proliferate. Reciprocal interaction and cross stimulation between MCPs producing vascular-endothelial growth factor (VEGF) and stromal cells producing IL-6 represent a potent regulatory mechanism contributing to increased vascularization in MM (74). Interestingly, OCLs are also participating to BM vascularization. As described for bone formation, bone resorption is coupled with angiogenesis mainly because OCLs produce and release VEGF from the bone matrix through the production of MMP9 (75, 76). Participation of OCLs to angiogenesis has also been reported in MM where osteopontin produced by OCLs cooperates with VEGF produced by MPCs to increase angiogenesis (77). Inhibition of OCL function reduces BM angiogenesis and tumor burden in mice (78).

**Figure 2:**
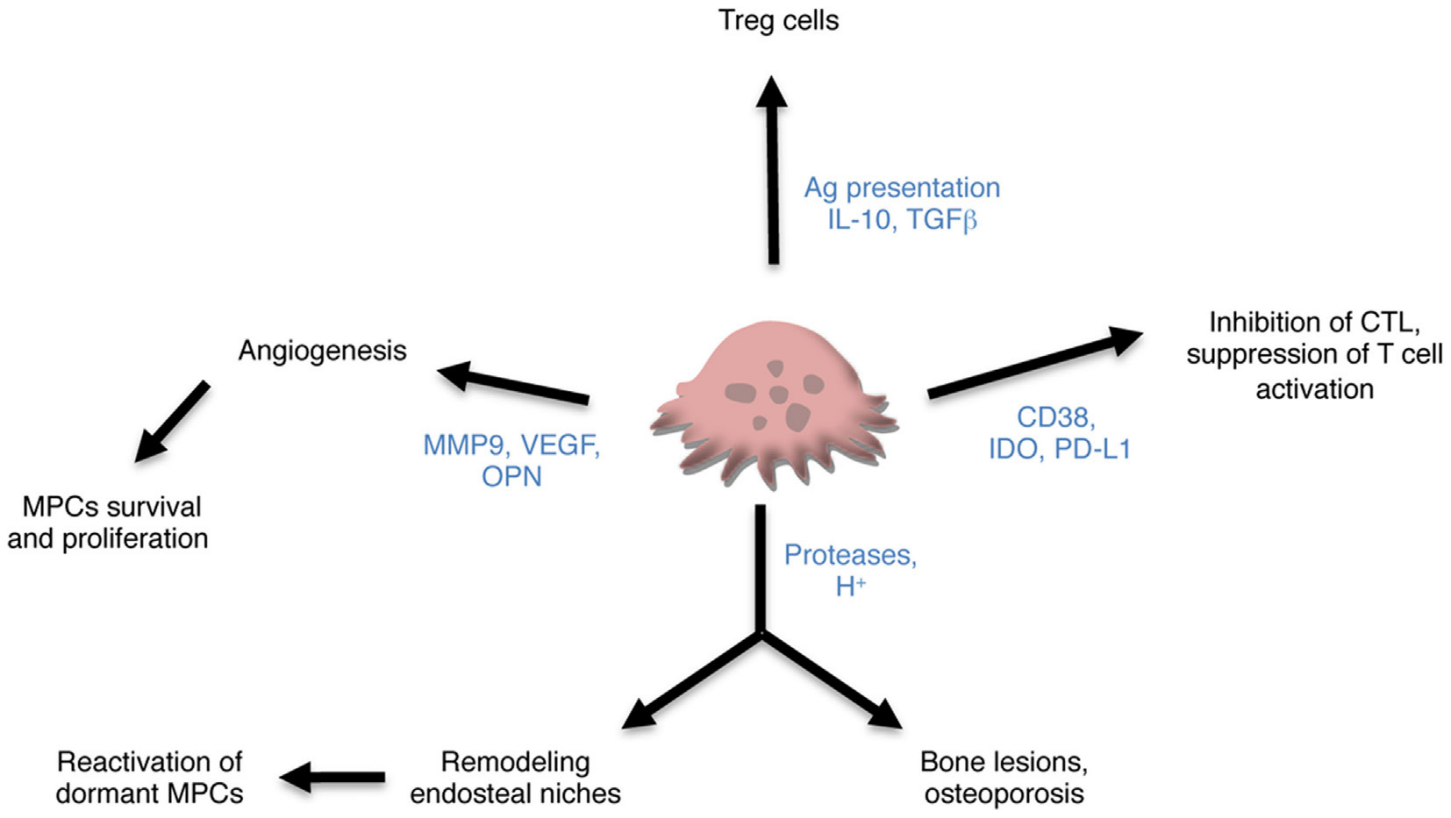
Contribution of osteoclasts (OCLs) to multiple myeloma (MM). OCLs may contribute to MM through different mechanisms. Producing proteases and proton, they are responsible not only for bone lesions but also for remodeling the endosteal niches and controlling the maintenance of dormant malignant plasma cells (MPCs). They also promote angiogenesis required for tumor cell survival and proliferation and tumor progression. Subsets of OCLs produce immunosuppressive cytokines and induce regulatory T (Treg) cells in an antigen-dependent manner. Lastly, OCLs express checkpoint proteins that participate in the inhibition of CTLs and suppression of T cell activation.

Overall, these data strongly support a key role of OCLs in the maintaining of MPCs in the BM through their control of BM niches and angiogenesis (Figure 2). How alteration in the BM niches participates in the progression of MGUS to SMM and MM is still a matter of research (79) and the role of OCLs in these alterations remains to determine.

## OCLs and Immunosuppressive Myeloma Environment

Tumor development is highly dependent on immune escape and impaired immune surveillance, including in MM (80). Crosstalk between MPCs and their environment favors the expansion of immune-suppressive cells and reduces the anti-tumor function of immune competent cells (81). Among these mechanisms, MM cells produce themselves IL-10 and other factors, such as ICOS-L that induce regulatory T (Treg) cells (82). In turn, Treg cells produce larger amount of immunosuppressive IL-10 and TGFβ than in healthy patients (83) and their proportion correlates with poor prognosis (84). NK cells are present in patients but have functional defects mediated by a high expression of programmed cell death 1 (PD-1) that binds to its ligand PD-L1 expressed on MM cells, participating to immune escape (85). MM cells also induce the development of myeloid-derived suppressor cells (MDSCs) that participate in the suppression of T cell responses and in the induction of Treg in part by producing arginase, reactive oxygen species, and nitric oxide (86–88). They also promote angiogenesis and support tumor growth (89). Moreover, MDSCs represent OCL progenitors and this potential is increased in MDSCs from MM patients (90).

While all these mechanisms have been largely documented, the contribution of OCLs to immunomodulation has been neglected for a long time. Nevertheless, OCLs derive from the myeloid lineage and share with other monocytic cells the capacity to modulate immune responses and interact with T cells as revealed by an increasing number of data including from our team (16, 19, 20, 70). Because they can arise from DCs, their capacity to process and present antigens has been evaluated. OCLs express MHC-II and the costimulatory molecules CD80 and CD86 *in vitro* (17, 19, 59) and *in vivo* (19) and process and present antigens (17, 19). In 2009, Kiesel et al. first reported that OCLs from healthy mice are able to cross present antigens inducing thereby the formation of CD8^+^ FoxP3^+^ Treg cells having an immunosuppressive function *in vitro* (16). In 2016, Ibanez et al. demonstrated that OCLs derived from normal BM express immunosuppressive cytokines (IL-10, TGFβ) and polarize CD4^+^ T cells toward immunosuppressive CD4^+^ Treg cells in an antigen-dependent manner (19). By contrast, OCLs derived from inflamed BM fail to induce Treg cells but instead they are very efficient in driving TNFα-producing CD4^+^ T cells (19). This study revealed for the first time the existence of different subsets of OCLs having opposite effect on T cell polarization depending on their cell origin and their environment (19).

In addition to this antigen-specific induction of different T cell subsets, OCLs have also been described as suppressive cells in steady state. Human OCLs derived from blood MN suppress T cell activation and proliferation independently from antigen presentation (18). Immune suppression by OCLs has also been reported in the context of MM. Many checkpoint molecules such as PD-L1, IDO HVEM, Galectin-9, and CD200 used by MPCs to escape immune surveillance are also expressed in OCLs from MM patients at a higher level than in MPCs and participate in T cell apoptosis or suppression (20). OCLs from patients protect MM cells against T cell cytolytic function through PD-L1 and IDO (20). OCLs also share with MCPs the expression of CD38. Treatment of OCLs with anti-CD38 antibodies downregulates expression of checkpoints proteins and reduces their immunosuppressive effect (20). These new findings on the participation of OCLs not only to bone lesion, but also to immune suppression point OCLs as key players in promoting MM development (Figure 2).

## Therapeutic Targeting of OCLs in MM

Over the past 15 years, identification of the impact of the microenvironment in cancer pathogenesis has transformed the therapeutic of MM with the develoment of drugs targeting the tumor in its microenvironment, such as proteasome inhibitors (PIs) and immunomodulatory drugs (IMiDs) (91, 92). These new therapies aim not only at inducing MPC apoptosis or blocking MPC proliferation but also at reducing angiogenesis and immunosuppression and at stimulating anti-tumoral responses. Interestingly, as OCLs are involved in many of these functions, these therapies also target the newly identified OCL functions.

By blocking the process of protein degradation necessary for many cellular functions mediated by proteasome, such as proliferation, activity, growth, and repair, PIs induce cellular apoptosis. Proliferating cancer cells, including myeloma cells, have a higher proteasome activity than normal cells and are more susceptible to PIs than normal cells. PIs have cytotoxic and growth inhibitory effects on MPCs (93–95) but they also display anti-osteoclastic, pro-osteoblastic, and anti-angiogenesis effects (96, 97). They inhibit the NF-κB and p38 MAP kinase pathways, they downregulate NFATc1, TRAF6, and the secretion of inflammatory cytokines, such as MIP-1α, BAFF, APRIL, IL-6, TNF-α, and IL-1β, all involved in osteoclastogenesis (98–103). Moreover, PIs increase the expression of BMP2, a potent inducer of OBL differentiation, and prevent the degradation of Runx2, the master gene of osteoblastogenesis, leading to new bone formation (104).

Immunomodulatory drugs have significant anti-myeloma activity (105). They induce MPC apoptosis, enhance anti-myeloma CTL and NK cell immunity and have antiangiogenic activity (106–110). Their mechanism of action includes caspase-8-mediated apoptosis, inhibition of the binding of MPCs to BM stromal cells, modulation of cytokine secretion, induction of immunogenic T, NK and NK-T cells, and downregulation of Treg cells (111). IMiDs also display anti-osteoclastogenic effects (99, 112) by affecting the lineage commitment of OCL precursors and downregulating critical factors involved in OCL differentiation and activity, such as PU.1, cathepsin K, and RANKL (99, 112, 113).

More recently, several anti-tumor mAbs have entered clinical testing in MM, inducing tumor cell death, reducing immune suppression, or stimulating anti-tumor immune responses. Because OCLs share the expression of many markers with MCP, they represent additional targets for these therapeutic approaches. Among these mAbs are the anti-CD38 mAbs (114). They induce myeloma cell death directly or through NK cell stimulation and complement-mediated cytotoxicity (115, 116). Interestingly, CD38 is highly expressed on OCLs and is involved in bone remodeling as evidenced by the reduced bone density observed in young CD38^−/−^ mice, a phenotype that disappears with age (25). CD38 is supposed to participate in the coupling between the OCL metabolism activity and calcium sensing, all required for bone resorption (25). In human, blocking of CD38 by anti-CD38 mAb inhibits *in vitro* the differentiation of MNs from MM patients into OCLs (117). Moreover, it reduces the immune suppressive activity of OCLs on T cell function by blocking the expression of immune checkpoint by OCLs (20).

Another example is the PD-1/PD-L1 axis, a master immune checkpoint regulating anti-tumor immune responses. PD-L1 is overexpressed in MDSCs, DC but also OCLs in the MM microenvironment. The binding between PD-L1 expressed on these cells and PD-1 on T lymphocytes results in the inhibition of T cell proliferation and cytokine secretion and in an increase in Treg cells resulting in immune suppression (118, 119). Blockade of this axis using an anti-PD-L1 Ab increases CD4^+^ T cell proliferation and CTL activity against human MM cells *in vitro* (120) and is curative in a murine model of myeloma in combination with antagonists of the IAP (inhibitors of apoptosis) proteins (121). These approaches are under evaluation in clinical trials. During their differentiation, OCLs upregulate immune inhibitory proteins, including PD-L1, that protect MPCs from immune responses. Thus OCLs are also likely to be targeted by these novel therapeutic strategies.

More specific approaches aim at blocking OCL differentiation and activity, such as bisphosphonates (BPs). Zoledronic acid or pamidronate are recommended for preventing skeletal-related events in patients with MM. However, they display side effects such as renal toxicity or osteonecrosis of the jaw (122). Novel inhibitors of OCL differentiation are under evaluation in MM, as Denosumab, a humanized anti-RANKL neutralizing antibody. Other molecules involved in bone destruction may represent interesting novel therapeutic targets. In particular, with the recent identification of the divergent immunomodulatory effects of the two OCL subsets (19), specific markers of these subsets should be identified and evaluated in preclinical studies to generate new therapeutic approaches specifically targeting each of these populations.

## Concluding Remarks

Despite the development of therapies targeting MPCs, MM remains largely incurable (123). The disease relapse is mainly due to immune escape and persistence in BM-specific niches of dormant MCPs that are resistant to therapy. Modifications of the cellular and molecular interactions in the BM during the course of the disease provide a microenvironment that favors these conditions. Thus, targeting MPCs is not sufficient and novel therapeutic strategies combining different targets, e.g., MCPs, immune checkpoints, and BM environment, are emerging (81).

In this sense, OCLs have long been considered only as responsible for bone lesions because of the pro-osteoclastogenic effect of MCPs. But as presented above, an increasing number of reports revealed that their function is not limited to bone resorption and that they may participate in modifications of BM niches, angiogenesis, myeloma cell maintenance, and immune suppression. Therefore, therapeutic approaches targeting OCLs probably affect not only bone resorption but also many other functions, and OCLs should not be considered anymore only as targets to improve the bone phenotype but also to improve immune responses.

Blocking bone resorption by BP or other inhibitors of bone resorption may be too limited to control all the detrimental effects of OCLs in myeloma. Moreover, MCP dormancy is a reversible state that can be switched “off” by OCLs (15). Thus, if inhibition of bone resorption is beneficial for bone lesions and reduces angiogenesis and tumor burden (78), it may also participate in the maintenance of MCP dormancy and resistance to therapy. Lastly, the identification of different OCLs subsets that induce immune tolerance or stimulate immunogenic responses revealed that targeting the harmful effects of OCLs in MM is probably much more complex than what has been envisaged up to now (19). A better understanding of the origin, function, and phenotype of the different OCL subsets is necessary to develop new approaches targeting specifically certain subsets of OCLs at certain phases of the disease, as done for other immune cells.

## Author Contributions

AM and CB-W wrote the manuscript. AM, CB-W, and AW reviewed the manuscript.

## Conflict of Interest Statement

The authors declare that their research was conducted in the absence of any commercial or financial relationships that could be construed as a potential conflict of interest.
